# Alexithymia and Empathy in Parent‐Youth Dyads: An Actor‐Partner Interdependence Model Analysis

**DOI:** 10.1002/pchj.70040

**Published:** 2025-07-22

**Authors:** Jia‐yi Zhou, Gui‐xiang Tian, Hai‐yue Li, Zi‐yu Wen, Ming‐yu Hu, Tong Yang, Neng‐zhi Jiang, Yi Wang, Yan‐yu Wang

**Affiliations:** ^1^ School of Psychology Shandong Second Medical University Weifang China; ^2^ Neuropsychology and Applied Cognitive Neuroscience Laboratory, CAS Key Laboratory of Mental Health Institute of Psychology, Chinese Academy of Sciences Beijing China; ^3^ Department of Psychology University of Chinese Academy of Sciences Beijing China

**Keywords:** actor‐partner interdependence model, alexithymia, empathy, parent‐youth dyads

## Abstract

Parent–child interaction plays a key role in the development and maintenance of individual social emotional ability. Although studies have found that parents' alexithymia affects their offspring's social–emotional abilities, it is unclear how parents' and children's alexithymia affect each other and their empathic abilities. This study examined the relationship between college students' and their parents' alexithymia and empathy, focusing on both actor effects (individual‐level associations) and partner effects (dyadic‐level associations). A total of 1058 parent‐youth dyads from a single college participated in the study, completing self‐report measures of alexithymia and empathy. Using an actor‐partner interdependence model analysis, the results revealed significant actor effects of alexithymia on cognitive empathy across all parent‐youth dyads, though no such effects were found for affective empathy. Additionally, significant partner effects were observed, with sons' alexithymia linked to their fathers' cognitive empathy and mothers' affective empathy. These findings emphasize the complex dynamics of social‐affective abilities within parent‐youth relationships among college students and provide important implications for future research, intervention, and prevention efforts.

## 1 Introduction

Alexithymia is a psychological condition characterized by an inability to recognize, describe, and understand one's and others' emotions, often described as “no words for feelings” (Picardi et al. 2005). Initially conceptualized to explain classic psychosomatic disorders (Nemiah 1976), alexithymia is now understood as a primary personality trait, affecting approximately 8% to 30% of the general population (Hamaideh 2018; Karukivi et al. 2010; McGillivray et al. 2017). Moreover, it has been identified as a risk factor for a range of mental health conditions, including depression, schizophrenia, autism spectrum disorder, and eating disorders (Beadle et al. 2013; Honkalampi et al. 2001; Mul et al. 2018; Yi et al. 2023). Beyond its intrapersonal consequences, alexithymia also impacts interpersonal relationships, with research indicating that individuals with high alexithymia tend to exhibit cold, distant social functioning and a detachment from others (De Rick and Vanheule 2006; Guttman and Laporte 2002). Clinical observations further suggest that alexithymia is associated with a distinctive social interaction style (Gerber et al. 2019; Grabe et al. 2001), indicating a poor capacity for socio‐affective skills.

Consistent with the aforementioned social behavior deficits, empirical work suggests that alexithymia is associated with diminished empathy, which is crucial for social awareness (Bird et al. 2010; Grynberg et al. 2010; Guttman and Laporte 2002; Moriguchi et al. 2007). Empathy is a multidimensional construct, comprising both affective and cognitive components (Bird and Viding 2014; Eisenberg 2000). Cognitive empathy refers to the capacity to understand and accurately perceive the emotional states of others, while affective empathy involves the ability to share and experience the emotions of others (Jackson et al. 2005; Jolliffe and Farrington 2004). According to introspection‐centric simulation theory (IST), alexithymia, as an internally oriented state, may contribute to empathy deficits, which are characterized by an externally oriented perspective (Valdespino et al. 2017). In one of the few studies examining the relationships between alexithymia and distinct domains of empathy, MacDonald and Price (2017) found significant negative correlations between alexithymia and cognitive empathy, but no significant association with affective empathy. This pattern suggests that impairments in emotional processing may specifically compromise individuals' ability to cognitively understand others' emotions, while leaving the capacity to experientially share others' affective states relatively unaffected. Given the limited and inconsistent evidence on the specific associations between alexithymia and distinct domains of empathy in the general population (Grynberg et al. 2010; Jonason and Krause 2013), additional research is needed to explore the nuances of these relationships.

Both individual and contextual factors play a crucial role in the development and maintenance of alexithymia and empathy, with the family serving as a key context for understanding the interplay between parent and child socio‐affective functioning (Daches et al. 2018; Guedeney and Dupong 2020). For instance, research has shown that children from highly expressive families tend to exhibit higher levels of emotional expressiveness, more frequent negative affect, and better nonverbal communication skills compared to children from less expressive families (Spitzer et al. 2009; Yelsma 2007). Additionally, young adults' perceptions of dysfunctional parental practices are strongly associated with higher levels of alexithymia (Barberis et al. 2023). Some studies also suggest that high levels of alexithymia in one parent are associated with lower levels of empathy in the other parent among adult women with borderline personality disorder (BPD) (Guttman and Laporte 2002). These findings suggest that the transmission of socio‐affective functioning within families is likely bidirectional, with mutual influences between parental and child processes shaping emotional and interpersonal outcomes across generations.

Another important area of focus within this field pertains to gender differences. Research consistently indicates that, compared to women, men tend to score higher on measures of alexithymia (Mendia et al. 2024) and lower on empathy (Rochat 2023). Clinical observations have further suggested that fathers of daughters with BPD are particularly prone to exhibiting alexithymic traits. Additionally, fathers of children with autism spectrum disorder (ASD) tend to exhibit lower empathy and higher alexithymia levels than mothers (Mensi et al. 2020). These findings highlight the need to consider gender roles when exploring the relationship between alexithymia and empathy in family contexts.

It is important to note that existing research on alexithymia and empathy within family contexts has largely overlooked the parent–child dyad, neglecting the reciprocal influence between parents and children. The actor‐partner interdependence model (APIM) offers a valuable framework for understanding how alexithymia in both parents and children contributes to their own (actor effect) and each other's (partner effect) empathy. Previous studies have applied APIM to explore family functioning and mental health in both parents and children (Milan et al. 2017; Wolff et al. 2020). However, no research has yet used this model to examine alexithymia and empathy in college students and their parents. Family members of college students, navigating the stage of emerging adulthood with tasks like separation‐individuation, are especially influenced during this period (McErlean and Lim 2020; Scharf and Goldner 2018). Therefore, APIM provides an innovative approach for examining the interaction between college students and their parents, offering a deeper understanding of the interpersonal dynamics of alexithymia and empathy within family contexts.

Despite growing recognition of alexithymia's role in socio‐affective functioning, a critical gap remains in understanding its dyadic manifestations within families. While previous research has established individual‐level associations between alexithymia and empathy, no study has systematically examined these relationships in parent‐youth dyads. The present study addresses this gap through three key contributions: (1) employing a dyadic approach to simultaneously assess intrapersonal (actor) and interpersonal (partner) effects, (2) examining potential gender‐specific patterns across different parent‐youth dyad configurations, and (3) distinguishing between cognitive and affective empathy dimensions. The primary aim of this study was to examine, using a parent‐youth dyadic approach, the intrapersonal and interpersonal associations between alexithymia and empathy. Specifically, this study investigates whether alexithymia in both college students (male and female) and their parents (father or mother) is related to their own empathy (cognitive or affective), as well as to their partner's empathy. Based on existing literature, it is hypothesized that higher levels of alexithymia would be significantly and negatively associated with both one's own empathy (actor effect) and their partner's empathy (partner effect). Furthermore, it is anticipated that these effects would be more pronounced in father‐son dyads. These investigations provide crucial insights into the relationships between alexithymia and empathy in family systems, guiding future interventions for socio‐affective difficulties.

## 2 Methods

### 2.1 Participants

Data were collected from an online survey of parents (*n* = 1937) and their children (*n* = 1594) at a university in Weifang, China. Informed consent was obtained electronically from all participants prior to completing the questionnaire. The study was approved by the Ethics Committee of Shandong Second Medical University. After removing responses with missing or inconsistent data, 1058 valid parent‐youth dyads were retained. The final sample consisted of 310 father‐son dyads (29.30%), 194 father‐daughter dyads (18.33%), 254 mother‐son dyads (24.01%), and 300 mother‐daughter dyads (28.36%). Parents' ages ranged from 38 to 69 years (*M* = 47.74, SD = 4.87 for fathers; *M* = 46.01, SD = 4.56 for mothers), while youths' ages ranged from 16 to 25 years (*M* = 18.78, SD = 0.96).

### 2.2 Measures

#### 2.2.1 Toronto Alexithymia Scale

The Chinese version of the Toronto Alexithymia Scale (Yao et al. 1991) was employed in this study to assess alexithymia. This self‐report questionnaire includes 26 items that assess three dimensions: difficulty identifying feelings, difficulty describing feelings, and externally oriented thinking. Participants rated each item on a 5‐point Likert scale, ranging from 1 (*strongly disagree*) to 5 (*strongly agree*), with higher scores indicating more pronounced alexithymic traits. The scale demonstrated good reliability and validity (Zhu et al. 2017).

#### 2.2.2 Questionnaire of Cognitive and Affective Empathy

The Chinese version of the Questionnaire of Cognitive and Affective Empathy (Liang et al. 2019) was used in this study to assess empathy. This self‐report questionnaire comprises 31 items that measure two dimensions: cognitive empathy and affective empathy. Participants rated each item on a 4‐point Likert scale, ranging from 1 (*strongly disagree*) to 4 (*strongly agree*), with higher scores indicating a greater level of empathy. The scale showed high internal consistency in this study, with alpha coefficients of 0.96 for cognitive empathy and 0.70 for affective empathy in parents, and 0.96 for cognitive empathy and 0.70 for affective empathy in youths.

## 3 Data Analysis

Descriptive statistics and correlations between parents' and youths' alexithymia and empathy were calculated in JASP for Windows, Version 0.19.1.0 (JASP Team 2021). The APIM analysis was conducted using APIM_SEM, a free web‐based tool that performs structural equation modeling with maximum likelihood estimation, utilizing the *lavaan* package in R (Stas et al. 2018). In our model, both parents' and youths' cognitive or affective empathy were included as dependent variables, while the total alexithymia scores of both parents and youths served as the independent variables. Moreover, demographic covariates, such as the age and gender of both the youths and their parents, were controlled for. Specifically, we examined the actor effect, which refers to the relationship between youths' alexithymia and their own cognitive or affective empathy, as well as the partner effect, which explores the influence of parents' alexithymia on their youths' cognitive or affective empathy. Additionally, we assessed the link between parents' alexithymia and their own cognitive or affective empathy (actor effect) and the relationship between youths' alexithymia and their parents' cognitive or affective empathy (partner effect). Our analysis focused on father‐son, father‐daughter, mother‐son, and mother‐daughter dyads.

## 4 Results

### 4.1 Descriptive Statistical Analysis

Descriptive statistics and Pearson's correlations for alexithymia, cognitive empathy, and affective empathy in father‐son, father‐daughter, mother‐son, and mother‐daughter dyads are presented in Tables 1, 2, 3, 4. Sons' alexithymia was negatively correlated with fathers' cognitive empathy. Sons' alexithymia was negatively correlated with mothers' affective empathy, and sons' cognitive empathy was negatively correlated with mothers' alexithymia. Daughters' alexithymia was negatively correlated with mothers' affective empathy.

**TABLE 1:** Correlations, means, and standard deviations for scores of alexithymia, cognitive and affective empathy in father‐son dyad.

Measure	1	2	3	4	5	6	M	SD
1. Sons alexithymia	—						73.20	7.41
2. Sons cognitive empathy	−0.23***	—					63.55	16.96
3. Sons affective empathy	−0.05	0.8***	—				33.16	7.95
4. Fathers alexithymia	−0.02	−0.04	0.00	—			74.67	7.26
5. Fathers cognitive empathy	−0.12*	0.03	−0.01	−0.24***	—		61.72	17.28
6. Fathers affective empathy	−0.08	−0.09	−0.08	−0.08	0.66***	—	32.33	6.02

*Note*: *p < 0.05, ***p < 0.001.

**TABLE 2:** Correlations, means, and standard deviations for scores of alexithymia, cognitive and affective empathy in father‐daughter dyad.

Measure	1	2	3	4	5	6	M	SD
1. Daughters alexithymia	—						68.62	9.53
2. Daughters cognitive empathy	−0.37***	—					66.98	13.00
3. Daughters affective empathy	−0.06	0.52***	—				33.87	6.39
4. Fathers alexithymia	0.07	−0.05	−0.05	—			70.77	9.25
5. Fathers cognitive empathy	−0.12	0.02	0.01	−0.34***	—		65.71	17.42
6. Fathers affective empathy	0.07	0.08	0.01	0.11	0.32***	—	32.42	6.95

*Note*: ***p < 0.001.

**TABLE 3:** Correlations, means, and standard deviations for scores of alexithymia, cognitive and affective empathy in mother‐son dyad.

Measure	1	2	3	4	5	6	M	SD
1. Sons alexithymia	—						71.75	8.26
2. Sons cognitive empathy	−0.25***	—					64.55	16.89
3. Sons affective empathy	0.11	0.62***	—				32.75	7.44
4. Mothers alexithymia	0.15*	−0.13*	−0.01	—			71.48	8.34
5. Mothers cognitive empathy	−0.10	0.06	0.03	−0.26***	—		63.12	18.00
6. Mothers affective empathy	−0.14*	−0.00	−0.08	0.08	0.47***	—	31.95	6.36

*Note*: *p < 0.05, ***p < 0.001.

**TABLE 4:** Correlations, means, and standard deviations for scores of alexithymia, cognitive and affective empathy in mother‐daughter dyad.

Measure	1	2	3	4	5	6	M	SD
1. Daughters alexithymia	—						67.65	9.82
2. Daughters cognitive empathy	−0.32***	—					69.27	13.04
3. Daughters affective empathy	0.12*	0.34***	—				33.77	6.86
4. Mothers alexithymia	−0.04	0.02	0.05	—			71.38	8.82
5. Mothers cognitive empathy	0.04	−0.09	−0.03	−0.25***	—		62.96	17.46
6. Mothers affective empathy	0.12*	−0.11	−0.03	−0.03	0.59***	—	32.74	6.25

*Note*: *p < 0.05, ***p < 0.001.

### 4.2 APIM Analysis for Alexithymia and Cognitive Empathy

**FIGURE 1:**
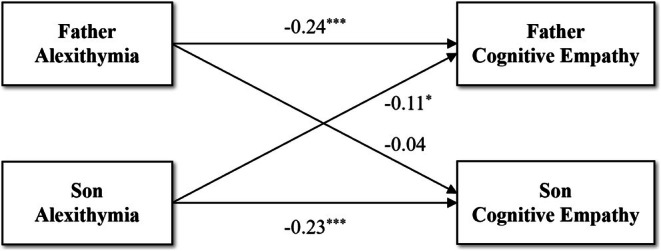
Actor‐Partner Interdependence Model of the relation between alexithymia and cognitive empathy in father‐son dyad. *p < 0.05, ***p < 0.001.

The results of the APIM for the relationship between alexithymia and cognitive empathy in the father‐son dyad are presented in Table 5 and Figure 1, and the other dyads are shown in Table S1 and Figures S1–S3. Regarding actor effects, significant actor effects were observed for all parent‐youth dyads, indicating that self‐reported levels of alexithymia are positively correlated with one's own rating of cognitive empathy. Additionally, a significant partner effect was only found in the father‐son dyad, revealing a negative association between sons' alexithymia and fathers' cognitive empathy.

**TABLE 5:** Actor‐partner interdependence model estimates for the relation between alexithymia and cognitive empathy in father‐son dyad.

Effect	Role	Estimate	95% CI	*p*	^*β*(o)	*r*
Intercept	Fathers	61.96	[60.09, 63.82]	< 0.001		
Actor		−0.58	[−0.83, −0.32]	< 0.001	−0.25	−0.24
Partner		−0.26	[−0.51, −0.01]	0.04	−0.11	−0.12
Intercept	Sons	63.23	[61.38, 65.08]	< 0.001		
Actor		−0.53	[−0,78, −0.29]	< 0.001	−0.10	−0.23
Partner		−0.10	[−0.36, 0.15]	0.43	−0.04	−0.05

Abbreviations: ^*β*(o), a standardized estimate using the overall standard deviation across both fathers and sons, which enables comparison of these estimates in father‐son dyad; CI, confidence interval; *r*, the partial correlation which provides the effect size for individual actor and partner effects.

### 4.3 APIM Analysis for Alexithymia and Affective Empathy

**FIGURE 2:**
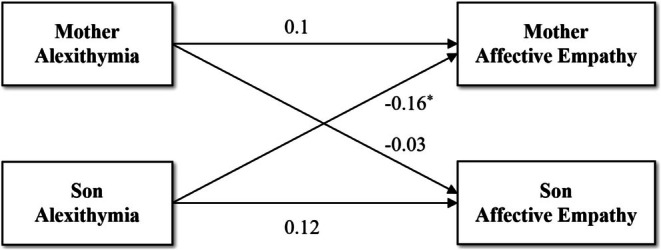
Actor‐Partner Interdependence Model of the relation between alexithymia and affective empathy in mother‐son dyad. *p < 0.05.

The results of the APIM for the relationship between alexithymia and affective empathy in mother‐son dyad are presented in Table 6 and Figure 2, and the other dyads are shown in Table S2 and Figures S4–S6. No significant actor effects were observed for any parent‐youth dyads, suggesting that self‐reported levels of alexithymia are not correlated with one's own rating of affective empathy. However, a significant partner effect was found only in mother‐son dyad, revealing a negative association between sons' alexithymia and mothers' affective empathy.

**TABLE 6:** Actor‐partner interdependence model estimates for the relation between alexithymia and affective empathy in mother‐son dyad.

Effect	Role	Estimate	95% CI	*p*	^*β*(o)	*r*
Intercept	Mothers	31.98	[31.21, 32.74]	< 0.001		
Actor		0.08	[−0.02, 0.17]	0.12	0.09	0.10
Partner		−0.12	[−0.22, −0.03]	0.01	−0.15	−0.16
Intercept	Sons	32.74	[31.83, 33.64]	< 0.001		
Actor		0.11	[−0.01, 0.22]	0.06	0.14	0.12
Partner		−0.02	[−0.13, 0.09]	0.69	−0.03	−0.03

Abbreviations: ^*β*(o), a standardized estimate using the overall standard deviation across both mothers and sons, which enables comparison of these estimates in mother‐son dyad; CI, confidence interval; *r*, the partial correlation that provides the effect size for individual actor and partner effects.

## 5 Discussion

The present study applied the APIM to explore individual and dyadic processes across different gender roles in the parent–child relationship. To the best of our knowledge, this is the first study to examine both the intrapersonal and interpersonal associations between alexithymia and empathy in youths and their parents. While both actor and partner effects were observed, the findings revealed some unexpected patterns. Specifically, expected actor effects were found for the relationship between alexithymia and cognitive empathy across all parent‐youth dyads, but not for alexithymia and affective empathy. Moreover, significant partner effects were identified in the context of sons' alexithymia and fathers' cognitive empathy, as well as sons' alexithymia and mothers' affective empathy. This study contributes to the existing literature by utilizing the APIM to investigate the interactions within the parent‐youth dyads among college students.

Our analysis revealed significant actor effects between alexithymia and cognitive empathy across all parent‐youth dyads, but no significant effects between alexithymia and affective empathy. This finding is notable given the emotional understanding and processing challenges faced by alexithymic individuals. However, it aligns with existing literature, which suggests stronger associations between alexithymia and cognitive empathy, compared to the more limited or weaker connections with affective empathy (Jonason and Krause 2013; MacDonald and Price 2017; New et al. 2012). Specifically, cognitive empathy was associated with multiple dimensions of alexithymia in the general population, whereas affective empathy was linked to only one dimension (Jonason and Krause 2013). Furthermore, clinical observations of individuals with BPD have shown that, while alexithymia often co‐occurs with deficits in cognitive empathy, affective empathy remains intact (New et al. 2012). Another study of college students also revealed that alexithymia was not related to affective empathy but mediated the relationship between mindfulness and cognitive empathy (MacDonald and Price 2017). Collectively, these findings suggest that cognitive and affective empathy may operate through distinct mechanisms, with cognitive empathy being more closely associated with alexithymia. These results are consistent with and support the introspection‐centric simulation theory, which posits that self‐awareness and attention to one's own emotional experiences are essential for understanding others' emotions, and that developing emotional awareness may facilitate empathy.

We observed a significant partner effect between sons' alexithymia and fathers' cognitive empathy, highlighting the interdependent association of these factors. In line with our findings, an fMRI study found reduced activity in the neural correlates of empathy in fathers of adolescents with ASD (Greimel et al. 2010). Additionally, a meta‐analysis showed that the broader autism phenotype (BAP), a collection of subclinical autistic traits, was more prevalent in parents of children with ASD than in control groups, with fathers exhibiting a higher prevalence than mothers (Rubenstein and Chawla 2018). Our results further suggested sons' alexithymia was directly associated with fathers' cognitive empathy in the general population. Notably, fathers often express more affection toward their young adult sons than sons do toward their fathers (Morman and Floyd 1999). Therefore, when sons live away at college, their limited social interactions with their fathers—combined with increased difficulties in emotional self‐expression—may reduce cognitive empathy in their fathers. However, we did not find a partner effect between fathers' alexithymia and sons' cognitive empathy. One possible explanation for this result is that, for college students, peer relationships may be more influential than paternal relationships in the development of social‐affective ability during this stage (Fraile et al. 2024; Zhou et al. 2020). Future research should further investigate this hypothesis to better understand these dynamics.

Further, it is noteworthy that no significant partner effect between alexithymia and cognitive empathy was observed among the female participants in the current study, except for the association between sons' alexithymia and mothers' affective empathy. This finding suggests that sex differences may play a crucial role in shaping the social‐affective functioning between individuals. Extensive developmental research has shown that sex differences in empathy emerge early in life and remain relatively stable across the lifespan (Michalska et al. 2013; Rochat 2023), with females consistently exhibiting higher levels of empathy than males. Another study has found that children who show higher levels of empathy early in development tend to maintain these traits as they grow (Eisenberg et al. 1999), suggesting that the female advantage in social‐affective ability may reflect an inherent evolutionary difference between males and females that is present from birth. Therefore, female cognitive empathy appears to be less susceptible to familial interactions compared to males.

In summary, the results in our study showed that alexithymia in sons negatively predicted both their father's cognitive empathy and their mother's emotional empathy. This pattern might reflect that male social‐affective processing is more sensitive to familial dynamics and interpersonal relationships. It could be that alexithymia in boys, who typically show lower levels of empathy compared to girls, amplifies challenges in understanding emotions, especially in the context of close family relationships. As a result, paternal cognitive empathy and maternal emotional empathy are particularly influenced by the son's emotional development, whereas this effect does not extend to daughters, likely due to their higher baseline levels of empathy, which may be less affected by familial interactions. Given the well‐documented sex differences in empathy (Rochat 2023), these findings underscore the need for more nuanced research on how gender interacts with familial factors to shape emotional and cognitive empathy across development. We suggest that the lack of such a predictive relationship in daughters might be attributed to the inherently higher levels of empathy typically exhibited by females, which could be less influenced by family dynamics.

A key theoretical contribution of this study is the use of the dyad as the unit of analysis, which allowed us to explore both intrapersonal and interpersonal relationships between alexithymia and empathy. Using the APIM approach, we uncovered the dynamic nature of these relationships. Our findings suggest that alexithymia is associated with cognitive empathy, but not affective empathy, advancing current theories on socio‐affective development. Additionally, the gender differences observed—such as the influence of sons' alexithymia on fathers' cognitive empathy and mothers' emotional empathy—underscore the importance of considering gender in future research on empathy development within families.

From a practical perspective, the findings suggest that interventions targeting cognitive empathy in both youths and parents could help mitigate the effects of alexithymia, particularly in families where this condition is prevalent. The observed gender differences also imply that interventions should be tailored for specific parent‐youth dyads, with father‐son interventions focusing on cognitive empathy and mother‐son interventions emphasizing emotional empathy. Future research adopting a similar dyadic framework will further enhance our understanding of family dynamics and socio‐affective development.

The findings of this study should be interpreted in light of several limitations. First, the cross‐sectional design prevents us from examining the temporal dynamics or causal relationships between youth and parent social‐affective ability. As such, it cannot capture how these abilities may influence one another over time. Longitudinal studies would be useful for investigating causal pathways and the transactional nature of these relationships. Second, because data were collected from only one parent per dyad, the study does not account for the interactions between both parents. This limits the ability to understand how family systems as a whole influence the development of social‐affective ability. Future research should include both parents in the analysis to provide a more comprehensive view of family dynamics. Third, the reliance on self‐report questionnaires introduces the possibility of response biases, such as social desirability or inaccurate self‐assessments. To address this, future studies should incorporate a range of methods, including experimental tasks and psychophysiological measures, to offer a more nuanced understanding of the relationship between alexithymia and empathy. Fourth, the lack of control for the number of siblings may influence parent‐youth interactions and the relationship between alexithymia and empathy. Research suggests that sibling dynamics significantly impact emotional development (Howe and Recchia 2014; Gungordu and Hernandez‐Reif 2022), and future studies should consider accounting for this factor.

In conclusion, the present study used an APIM analysis to explore the relationships between alexithymia and empathy among youths and their parents. The findings showed that both youths' and parents' alexithymia was associated with their own cognitive empathy across all parent‐youth dyads, but not affective empathy. Additionally, sons' alexithymia was linked to their fathers' cognitive empathy and mothers' affective empathy. These results contribute to a deeper theoretical understanding of the interplay between various social‐affective abilities within family contexts and offer important implications for future research, intervention, and prevention efforts.

## Conflicts of Interest

The authors declare no conflicts of interest.

## Supporting information


**Figure S1.** Actor‐Partner Interdependence Model of the relation between alexithymia and cognitive empathy in father‐daughter dyad. ***p < 0.001.
**Figure S2.** Actor‐Partner Interdependence Model of the relation between alexithymia and cognitive empathy in mother‐son dyad. ***p < 0.001.
**Figure S3.** Actor‐Partner Interdependence Model of the relation between alexithymia and cognitive empathy in mother‐daughter dyad. ***p < 0.001.
**Figure S4.** Actor‐Partner Interdependence Model of the relation between alexithymia and affective empathy in father‐son dyad.
**Figure S5.** Actor‐Partner Interdependence Model of the relation between alexithymia and affective empathy in father‐daughter dyad.
**Figure S6.** Actor‐Partner Interdependence Model of the relation between alexithymia and affective empathy in mother‐daughter dyad. *p < 0.05.
**Table S1.** Actor‐Partner Interdependence Model estimates for the relation between alexithymia and cognitive empathy in father‐daughter, mother‐son, mother‐daughter dyads.
**Table S2.** Actor‐Partner Interdependence Model estimates for the relation between alexithymia and affective empathy in father‐son, father‐daughter, mother‐daughter dyads.
