# Supplementary material for: Alexithymia and Empathy in Parent‐Youth Dyads: An Actor‐Partner Interdependence Model Analysis
Source: Psych J. 2025 Jul 22;14(5):650–7. doi: 10.1002/pchj.70040 (PMC12520845; doi:10.1002/pchj.70040)
Supplement: Supplementary file 1 — Figure S1. Actor‐Partner Interdependence Model of the relation between alexithymia and cognitive empathy in father‐daughter dyad. ***p < 0.001. Figure S2. Actor‐Partner Interdependence Model of the relation between alexithymia and cognitive empathy in mother‐son dyad. ***p < 0.001. Figure S3. Actor‐Partner Interdependence Model of the relation between alexithymia and cognitive empathy in mother‐daughter dyad. ***p < 0.001. Figure S4. Actor‐Partner Interdependence Model of the relation between alexithymia and affective empathy in father‐son dyad. Figure S5. Actor‐Partner Interdependence Model of the relation between alexithymia and affective empathy in father‐daughter dyad. Figure S6. Actor‐Partner Interdependence Model of the relation between alexithymia and affective empathy in mother‐daughter dyad. *p < 0.05. Table S1. Actor‐Partner Interdependence Model estimates for the relation between alexithymia and cognitive empathy in father‐daughter, mother‐son, mother‐daughter dyads. Table S2. Actor‐Partner Interdependence Model estimates for the relation between alexithymia and affective empathy in father‐son, father‐daughter, mother‐daughter dyads. [file PCHJ-14-650-s001.docx]

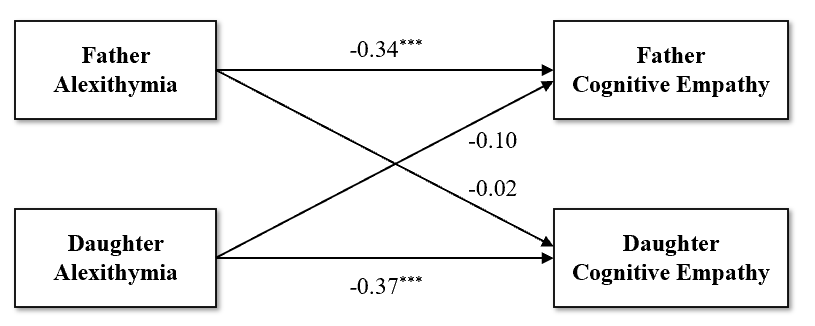


FIGURE S1 Actor-Partner Interdependence Model of the relation between alexithymia and cognitive empathy in father-daughter dyad. ****p < 0.001.*


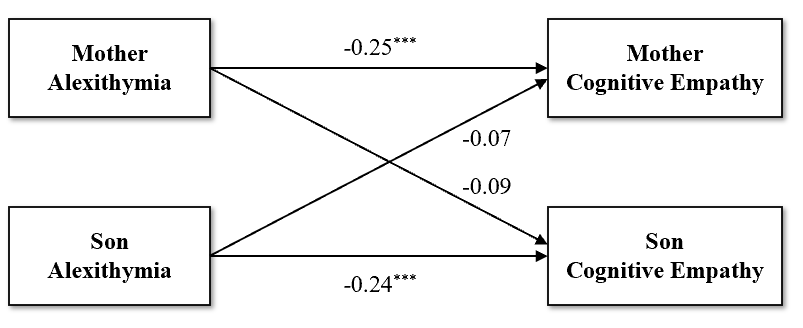


FIGURE S2 Actor-Partner Interdependence Model of the relation between alexithymia and cognitive empathy in mother-son dyad. ****p < 0.001.*


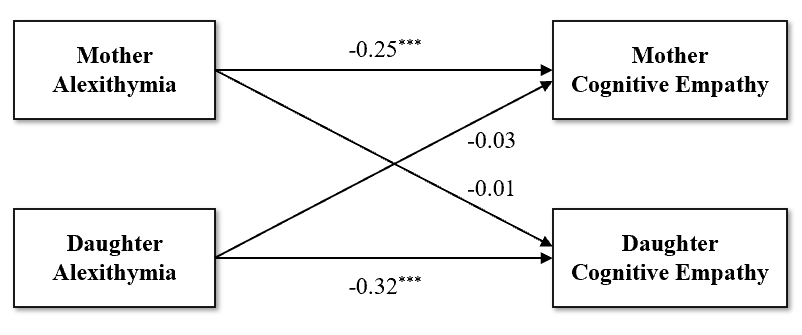


FIGURE S3 Actor-Partner Interdependence Model of the relation between alexithymia and cognitive empathy in mother-daughter dyad. ****p < 0.001.*


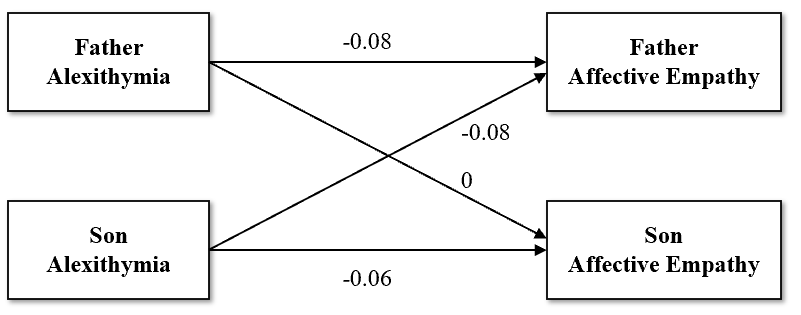


FIGURE S4 Actor-Partner Interdependence Model of the relation between alexithymia and affective empathy in father-son dyad.


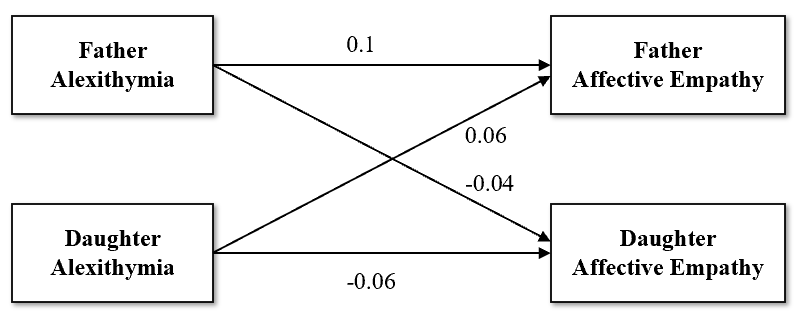


FIGURE S5 Actor-Partner Interdependence Model of the relation between alexithymia and affective empathy in father-daughter dyad.


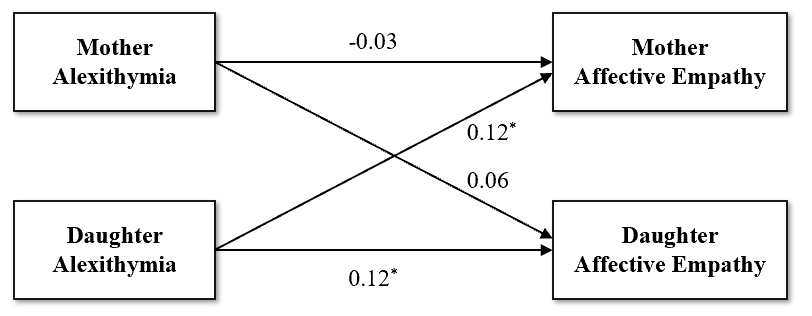


FIGURE S6 Actor-Partner Interdependence Model of the relation between alexithymia and affective empathy in mother-daughter dyad. **p < 0.05.*

TABLE S1 Actor-Partner Interdependence Model estimates for the relation between alexithymia and cognitive empathy in father-daughter, mother-son, mother-daughter dyads.

| Effect | Role | Estimate | 95% CI | p-value | ^β(o) | r |
| --- | --- | --- | --- | --- | --- | --- |
| Intercept | fathers | 66.20 | [63.89, 68.52] | <.001 |  |  |
| Actor |  | -0.63 | [-0.88, -0.38] | <.001 | -0.39 | -0.34 |
| Partner |  | -0.18 | [-0.42, 0.07] | 0.15 | -0.11 | -0.10 |
| Intercept | daughters | 66.47 | [64.75, 68.18] | <.001 |  |  |
| Actor |  | -0.50 | [-0.69, -0.33] | <.001 | -0.23 | -0.37 |
| Partner |  | -0.03 | [-0.22, 0.15] | 0.73 | -0.02 | -0.02 |
| Intercept | mothers | 63.06 | [60.94, 65.19] | <.001 |  |  |
| Actor |  | -0.55 | [-0.81, -0.29] | <.001 | -0.26 | -0.25 |
| Partner |  | -0.14 | [-0.40, 0.12] | 0.29 | -0.07 | -0.07 |
| Intercept | sons | 64.586 | [62.59, 66.58] | <.001 |  |  |
| Actor |  | -0.49 | [-0.73, -0.24] | <.001 | -0.11 | -0.24 |
| Partner |  | -0.19 | [-0.43, 0.05] | 0.13 | -0.09 | -0.10 |
| Intercept | mothers | 63.96 | [61.98, 65.94] | <.001 |  |  |
| Actor |  | -0.49 | [-0.70, -0.27] | <.001 | -0.29 | -0.25 |
| Partner |  | 0.05 | [-0.14, 0.25] | 0.60 | 0.03 | 0.03 |
| Intercept | daughters | 68.47 | [67.02, 69.91] | <.001 |  |  |
| Actor |  | -0.42 | [-0.57, -0.28] | <.001 | -0.19 | -0.32 |
| Partner |  | 0.01 | [0.15, 0.17] | 0,91 | 0.01 | 0.01 |

Abbreviations: CI = confidence interval; ^β(o) = a standardized estimate using the overall standard deviation across both parent-youth dyads, which enables comparison of the estimates across these dyads; r = the partial correlation which provides the effect size for individual actor and partner effects.

TABLE S2 Actor-Partner Interdependence Model estimates for the relation between alexithymia and affective empathy in father-son, father-daughter, mother-daughter dyads.

| Effect | Role | Estimate | 95% CI | p-value | ^β(o) | r |
| --- | --- | --- | --- | --- | --- | --- |
| Intercept | fathers | 32.33 | [31.66, 33.00] | <.001 |  |  |
| Actor |  | -0.07 | [-0.13, 0.03] | 0.33 | -0.07 | -0.08 |
| Partner |  | -0.06 | [-0.15, 0.03] | 0.96 | -0.07 | -0.08 |
| Intercept | sons | 33.11 | [33.22, 34.00] | <.001 | -0.06 | -0.06 |
| Actor |  | -0.06 | [-0.18, 0.06] | 0.15 | 0.00 | 0.00 |
| Partner |  | 0.00 | [-0.12, 0.12] | 0.17 |  |  |
| Intercept | fathers | 32.38 | [31.40, 33.37] | <.001 |  |  |
| Actor |  | 0.08 | [-0.03, 0.18] | 0.15 | 0.11 | 0.10 |
| Partner |  | 0.04 | [-0.05, 0.15] | 0.40 | -0.04 | 0.06 |
| Intercept | daughters | 33.86 | [32.95, 34.76] | <.001 |  |  |
| Actor |  | -0.04 | [-0.13, 0.06] | 0.42 | -0.08 | -0.06 |
| Partner |  | -0.03 | [-0.13, 0.07] | 0.53 | -0.04 | -0.05 |
| Intercept | mothers | 32.92 | [32.19, 33.65] | <.001 |  |  |
| Actor |  | -0.02 | [-0.10, 0.06] | 0.66 | -0.03 | -0.03 |
| Partner |  | 0.08 | [0.01, 0.15] | 0.04 | 0.11 | 0.12 |
| Intercept | daughters | 33.85 | [33.05, 34.65] | <.001 |  |  |
| Actor |  | 0.09 | [0.01, 0.16] | 0.03 | 0.17 | 0.12 |
| Partner |  | 0.04 | [-0.04, 0.13] | 0.34 | 0.06 | 0.06 |

Abbreviations: CI = confidence interval; ^β(o) = a standardized estimate using the overall standard deviation across both parent-youth dyads, which enables comparison of the estimates across these dyads; r = the partial correlation which provides the effect size for individual actor and partner effects.
